# Dermoid and epidermoid cysts of the oral cavity: a 48-year retrospective study with focus on clinical and morphological features and review of main topics

**DOI:** 10.4317/medoral.23388

**Published:** 2020-03-06

**Authors:** Hellen Bandeira de Pontes Santos, Larissa Santos Amaral Rolim, Caio César da Silva Barros, Israel Leal Cavalcante, Roseana de Almeida Freitas, Lélia Batista de Souza

**Affiliations:** 1 DDS, MSc, PhD in Oral Pathology, Postgraduate Program in Dental Sciences, Department of Dentistry, Federal University of Rio Grande do Norte, Natal, RN, Brazil; 2 DDS, MSc, PhD Student of Oral Pathology and Medicine, Postgraduate Program in Dental Sciences, Department of Dentistry, Federal University of Rio Grande do Norte, Natal, RN, Brazil; 3 DDS, MSc in Oral Pathology, Professor, Department of Dentistry, University of Fortaleza, Fortaleza, CE, Brazil; 4 DDS, MSc, PhD in Oral Pathology, Professor, Postgraduate Program in Dental Sciences, Department of Dentistry, Federal University of Rio Grande do Norte, Natal, RN, Brazil

## Abstract

**Background:**

Dermoid and epidermoid cysts are slow-growing, benign developmental cysts that arise from ectodermal tissue and can occur anywhere in the body. Less than 7% of these cysts involve the head and neck region, with only 1.6% of cases presenting in the oral cavity. To evaluate the clinical and histopathological features of dermoid (DCs) and epidermoid (ECs) cysts stored in the archives of a referred Oral Pathology Service over a 48-year-period, and to review current concepts about these cysts.

**Material and Methods:**

All DCs and ECs were reviewed, and clinical data were obtained from the patient records. Fourteen cases of DCs and thirteen cases of ECs were re-evaluated microscopically by 2 oral pathologists.

**Results:**

Among 15.387 cases, 14 (0.09%) had a histopathological diagnosis of DCs and 13 (0.08%) of ECs. For DCs, ten (71.4%) patients were women, with the mean age of 37.2 years. All DCs were lined by a stratified squamous epithelium (100%), with gut and respiratory epithelium observed in 1 (7.1%) and 2 (14.3%) cases, respectively. Chronic inflammatory cells, melanin, multinucleated giant cell reaction, and Pacini bodies were also observed. For ECs, eight (61.5%) cases were in women, and the mean age was 38.2 years. All ECs were lined by a stratified squamous epithelium (100%). Chronic inflammatory cells, melanin pigmentation, and adipose tissue were observed in the fibrous capsule.

**Conclusions:**

Our results suggest that stratified squamous epithelium is the predominant epithelial lining of these cystic lesions. Also, we may find some unusual findings in DCs, such as Pacini bodies.

** Key words:**Non-odontogenic cysts, epidermoid cysts, dermoid cysts, diagnosis.

## Introduction

Dermoid and epidermoid cysts (DCs and ECs, respectively) are slow-growing, benign developmental cysts that arise from ectodermal tissue and can occur anywhere in the body (1,2). They are most commonly located in places where embryonic elements merge, especially in the sacral region and ovaries, while less than 7% of these cysts involve the head and neck region, with only 1.6% of cases presenting in the oral cavity (2-4). In this regard, the floor of the mouth is the second most common head and neck region site after the lateral eyebrow region, since these represent embryonic fusion sites. However, these cysts can also be found on the tongue, lips and other oral mucosa locations (3,4).

ECs are the most common, and several mechanisms have been proposed for their formation, such as the proliferation of ectodermal remnants during embryogenesis, obstruction of pilosebaceous units or traumatic implantation of epithelial cells (5,6). In relation to DCs, these appear to be derived from epithelial remnants included during the union of the midline of the first and second gill arches (3,4,7). In addition, dysonogenic, traumatic and thyroglossus anomaly theories have been postulated as being responsible for their pathogenesis (3,4,7).

Despite their congenital origin, these cysts are often diagnosed in the second and third decades of life (3,7), clinically presenting as painless swellings which, in some cases, may interfere with phonation, chewing or swallowing (6,7).

ECs are always coated by keratinized stratified squamous epithelium without dermal appendages within the underlying fibrous connective tissue. DCs present, in addition to ECs characteristics, dermal appendages such as hair follicles, hairs and sebaceous and sweat glands (3,4,6,7).

DCs and ECs can be present in approximately 60% of cases of hereditary familial polyposis and Gardner’s syndrome. In these cases, the cysts are seen most frequently on the face, on the scalp, the arms and legs (8). Malignant transformation of dermoid and epidermoid cysts is exceptionally rare, with a 5% rate, and it has previously been described in the head and neck, ovarian, intracranial, and lumbar region (2-4,6).

Retrospective studies that address the clinicopathological aspects of ECs and DCs are essential in order to clarify divergences about their clinical and histopathological characteristics, as well as provide further epidemiological information. Therefore, the aim of the present study was to retrospectively describe the clinical and pathological characteristics of DCs and ECs diagnosed at a Pathological Anatomy Service in the Brazilian Northeast throughout 48 years, emphasizing their varied histopathological characteristics. A literature review on these lesions was also carried out.

## Material and Methods

All cases of DCs and ECs diagnosed between January 1970 and March 2018 retrieved from the Oral Pathology Service archive of UFRN were reviewed. This service is one of the referral centers in oral and maxillofacial pathology in Brazil. Data such as patient age, sex, anatomical site, clinical aspect, time of the lesion, size and treatment were compiled for all cases from the clinical data sent together with the biopsy records.

For histopathologic analysis, all slides containing hematoxylin/eosin-stained 5-µm-thick sections were reassessed. Dermoid and epidermoid cysts were histologically reviewed by two trained examiners under a light microscope (Olympus CX31, Olympus Japan Co., Tokyo, Japan). Histological findings of the cysts were classified according to the morphological features described by Shear and Speight (1), such as the type of epithelium (squamous stratified, gut epithelium, and respiratory epithelium). For dermoid cysts, it was analyzed the presence of fair follicle, hair, sweat gland, sebaceous gland, and salivary gland. It was also analyzed features of the cystic wall (neural tissue, smooth and striated muscle, adipose tissue, bone, cartilage, and inflammatory infiltrate). The presence of the following morphological findings was also considered: negative spaces of cholesterol crystals, multinucleated giant cell reaction, hyaline ring granulomas, Pacini bodies, Meissner bodies, and melanin pigmentation.

The data were tabulated and analyzed by descriptive statistics using the IBM SPSS Statistics program (version 20.0; IBM Corp., Armonk, NY, USA).

## Results

There were 15.387 cases of oral and maxillofacial lesions during the period studied; of these, 14 (0.09%) had a histopathological diagnosis of dermoid cyst and 13 (0.08%) of epidermoid cyst. Only cases located in the oral cavity were selected and reassessed for this study.

- Dermoid cysts

For dermoid cysts, there was a higher frequency of dermoid cysts in women (n = 10, 71.4%) (female: male ratio of 2.5:1). The age of the patients ranged from 1 to 81 years, with a mean of 37.2 years. Most cases occurred in the floor of the mouth (n = 6, 42.9%) and lip (n = 6, 42.9%), being five on the lower lip and one case on the upper lip (Table 1). The cysts appeared as asymptomatic nodules and the mean size was 3.3 ± 3.3cm with an average duration of 53.3 months, ranging from 1 to 300 months (Table 1).

**Table 1:**
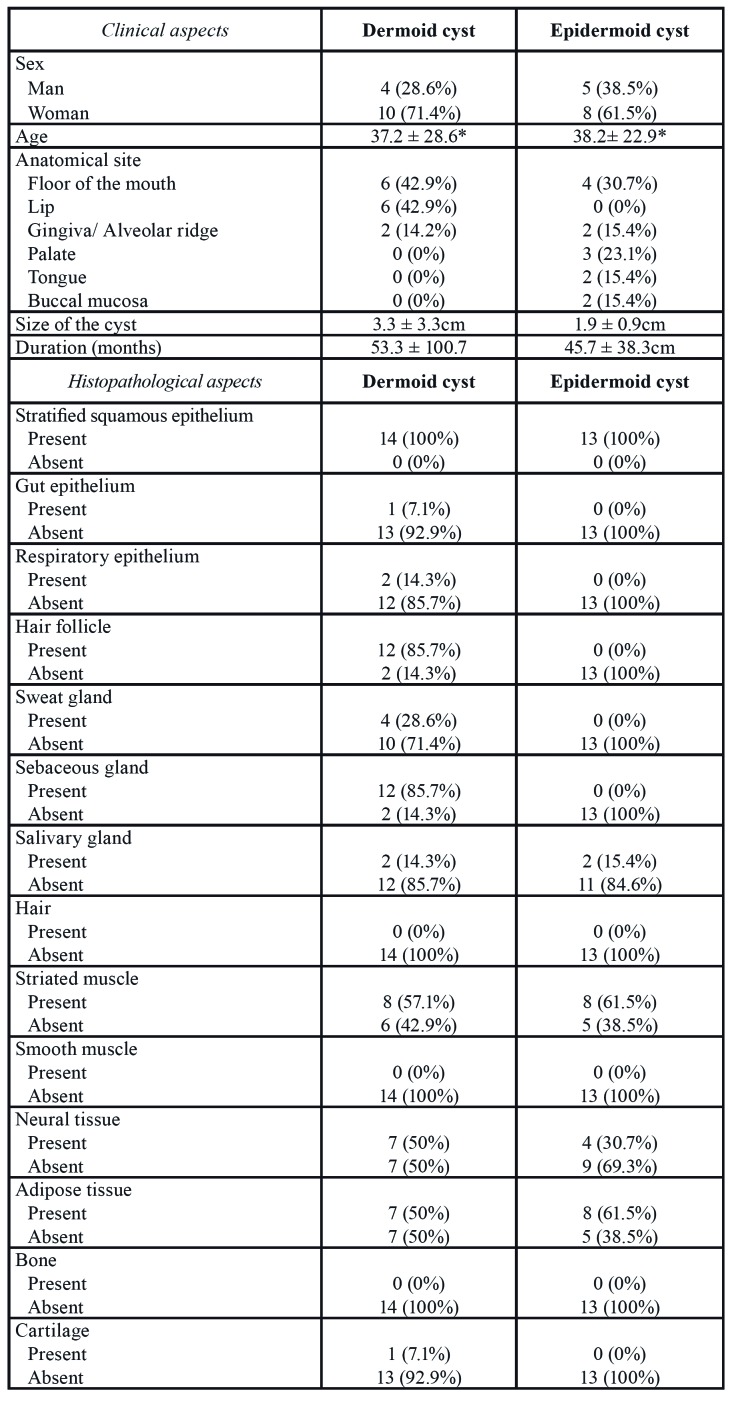
Frequency of clinical and histopathological aspects of dermoid and epidermoid cyst from the present study.

**Table 1 cont.**
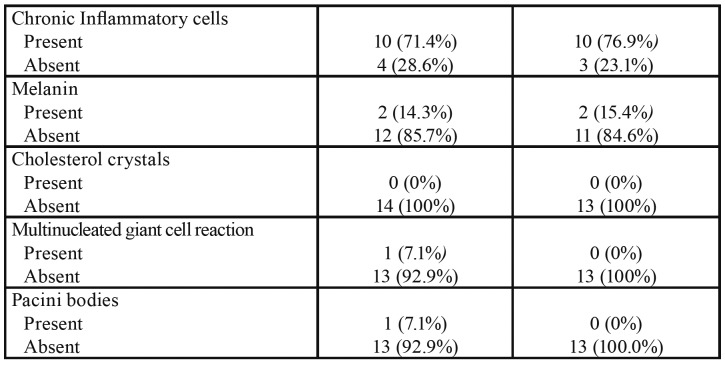
Frequency of clinical and histopathological aspects of dermoid and epidermoid cyst from the present study.

**Figure 1:**
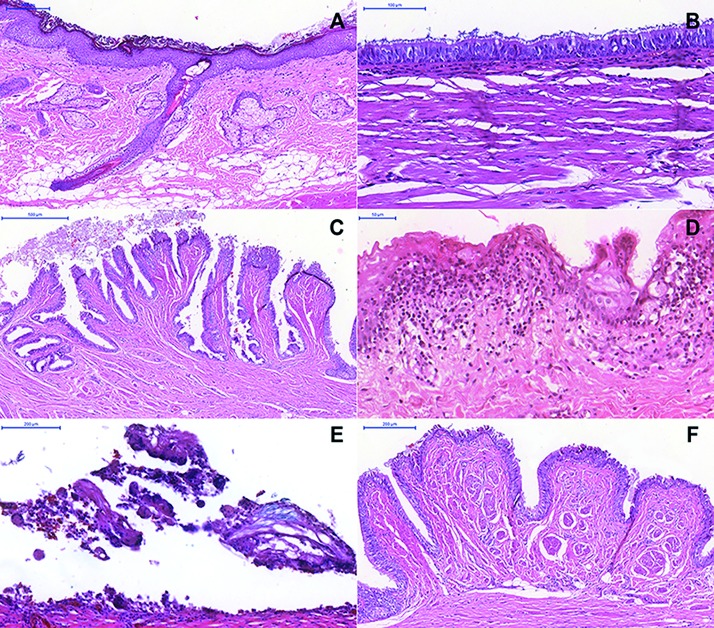
Histopathological features of dermoid cysts (DCs) (Hematoxylin & Eosin) – (A) Cystic lesion lined by stratified squamous epithelium and hair follicle, sweat gland, sebaceous gland, and adipose tissue in the fibrous capsule (Scale bars = 200 µm). (B) Ciliated pseudostratified columnar (Scale bars = 100 µm) and (C) gut epithelium lining (Scale bars = 500 µm). (D) Chronic inflammatory cells (Scale bars = 50 µm) and (E) multinucleated giant cells (Scale bars = 200 µm). (F) Pacini bodies (Scale bars = 200 µm).

Microscopically, all dermoid cysts were lined by a stratified squamous epithelium (Fig. 1) (100%). Respiratory and gut epithelium was observed in 2 (14.3%) (Fig. 1) and 1 (7.1%) (Fig. 1) cases, respectively (Table 1). In the fibrous capsule, eight cases had striated muscle (57.1%), any case had smooth muscle (0.0%), seven cases had neural tissue (50.0%), seven cases presented adipose tissue (50.0%), any case had bone tissue (0.0%), and one case presented cartilage (7.1%). Chronic inflammatory cells (Fig. 1), melanin, multinucleated giant cell reaction (Fig. 1), and Pacini bodies (Fig. 1) were found in 10 (71.4%), 2 (14.3%), 1 (7.1%), 1 (7.1%) of the cases, respectively. Lymphoid follicle and negative spaces of cholesterol crystal were not observed in the dermoid cysts.

- Epidermoid cysts

Among the epidermoid cysts, eight were diagnosed in women (61.5%) with a female:male ratio of 1.6:1. The age of the patients ranged from 11 months to 82 years, with a mean of 38.2 years. Most cases occurred in the floor of the mouth (n = 4, 30.7%), followed by palate (n = 3, 23.1%). The cysts appeared as asymptomatic nodules in the oral mucosa and the mean size of lesions was 1.9 ± 0.9cm with an average duration of 45.7 months, ranging from 2 to 120 months (Table 1).

**Figure 2:**
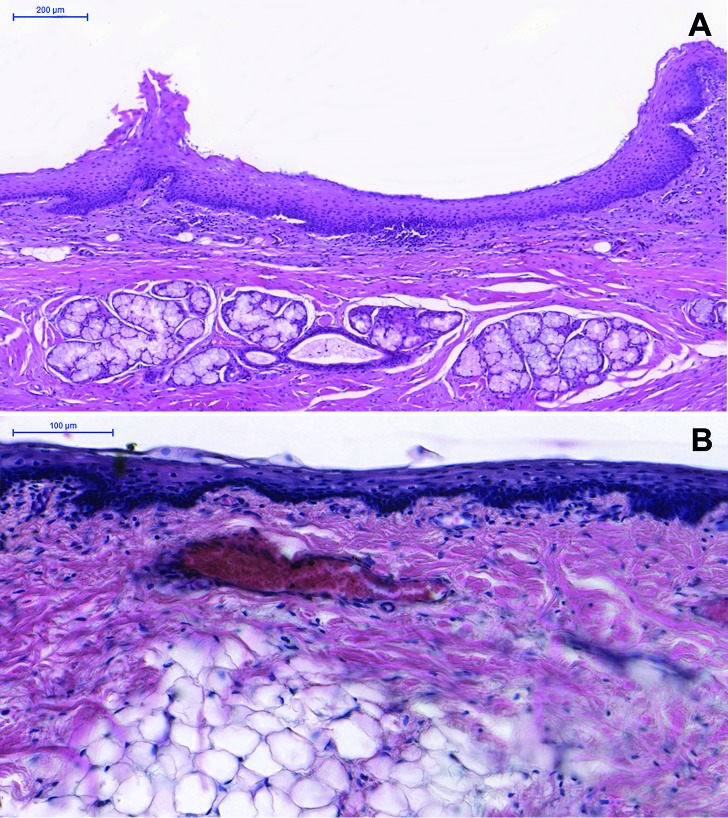
Histopathological features of epidermoid cysts (ECs) (Hematoxylin & Eosin) – Cystic lesion lined by stratified squamous epithelium with (A) salivary gland and (B) adipose tissue in the cystic capsule (Scale bars = 200 µm and 100 µm, respectively).

With respect to microscopical findings, all epidermoid cysts were lined by a stratified squamous epithelium (Fig. 2) (100%). In the fibrous capsule, two cases had salivary glands (Fig. 2) (15.4%), eight cases had adipose tissue (Fig. 2) (61.5%), two cases presented melanin pigmentation (15.4%), and one case had multinucleated giant cell reaction (7.1%). Smooth muscle, bone tissue, cartilage, and Pacini bodies associated to the cyst were not observed in our cases.

## Discussion

DCs and ECs are uncommon lesions in the head and neck region, corresponding to 7% of all cases, presenting even less frequently in the oral cavity, representing between only 0.01% and 1.6% of all oral cysts (3,4,9-11). Yilmaz *et al*. (11) report that ECs are more common when compared to DCs. This retrospective study was performed at an oral diagnosis reference center in the Brazilian northeast, where, from a total of 15,387 diagnosed cases, only 14 (0.09%) were diagnosed as DC and 13 (0.08%) as EC throughout 48 years, with no difference in prevalence between both types.

Some studies report that DCs and ECs present similar distribution between genders (4,12,13), with higher frequency in individuals between 15 and 35 years old (4,12,14,15). Similarly, to what is reported in the literature, DCs exhibited a higher frequency in females, whereas ECs display only a discrete predilection. In addition, both cysts were detected in individuals with mean age close to 35 years old (37.2 ± 28.6 and 38.2 ± 22.9, respectively). However, it is important to point out that these lesions can be diagnosed in a wide age group, with reports as distinct as in 7-month and 77-year-old individuals (9).

The clinical presentation of these lesions in the oral cavity depends on their size, which can vary between 1 and 5 cm, as well as their anatomical location (16). DCs and ECs appear as asymptomatic nodules mainly affecting the floor of the mouth but may also affect other regions such as the lips, jugal mucosa and tongue (3,4,10,14,15). In the cases analyzed herein, the most affected anatomic site was the floor of the mouth, followed by the lip for DCs and by the palate for ECs. In addition, the mean DCs size was of 3.3 ± 3.3 cm, while ECs size was of 1.9 ± 0.9 cm, corroborating literature reports.

The histopathological characteristics of DCs and ECs are variable, with the observation of stratified squamous epithelium coatings in both. If no dermal attachments are present, the cyst is called epidermoid. If attachments, such as sweat glands, sebaceous glands and hair follicles, are present, the cyst is referred to as dermoid. In the present study, the histological characteristics and classifications proposed by Shear and Speight (1) were used for the histological evaluation of DCs and ECs. For DCs, a stratified squamous epithelial lining was observed in all cases (100.0%). In two cases (14.3%), respiratory epithelium areas were observed, followed by one case presenting intestinal epithelium areas (7.1%). As noted in the present study, DCs may exhibit variability in epithelial types. Thus, pathologists should be aware of these characteristics and should always associate them with clinical findings in order to establish the correct diagnosis.

DCs present a pathological cavity lined by epithelium, displaying skin attachments like hair, hair follicles, sebaceous glands, and sweat glands on the cystic wall (17). Keratinous or sebaceous material may be present within the cystic space. In some cases, a foreign body giant cell reaction may result due to cyst rupture, leading to cystic content spillage (17). Several morphological findings are often seen on the cystic wall of DCs. The presence of hair follicles and sebaceous glands were observed in most cases (85.7%). Other findings, such as sweat glands, salivary glands, melanin pigments, and Pacini corpuscles, were also noted, observed in 28.6%, 14.3%, 14.0%, 7.1% e 7.1% of the cases, respectively. Other structures in the cystic wall have been described, such as hair and cholesterol clefts (17). However, we did not detect these microscopic findings in the cases analyzed herein.

Microscopically, DCs can be differentiated from ECs by the presence of cutaneous appendages, and from teratomas by a lack of tissues originating from different germ layers, such as cartilage (14).

Surgical excision is the treatment of choice for both cysts and can be performed by an intra- or extraoral approach, which is chosen from the location and size of the lesion (3,4,10). The intraoral approach is generally chosen for small sublingual cysts, superior to the mylohyoid muscle, while the extraoral approach is preferred for large and cysts inferior to muscle (4). DCs and ECs recurrence is rare, since the presence of the fibrous capsule facilitates cyst enucleation (15).

## Conclusions

DCs and ECs are relatively uncommon lesions in the oral cavity. The clinical characteristics of the DCs and ECs observed in the present study are similar to those reported in the literature. Regarding histopathological features, the results reported herein indicate that the epithelial lining, commonly found in both DCs and ECs, comprises stratified squamous epithelium. DCs may exhibit various structures in the middle of the fibrous capsule, with cutaneous attachments as the most frequent. However, although they are less frequent, Pacini corpuscles can also be found in DCs.
